# Nanoplastic Impact on the Gut-Brain Axis: Current Knowledge and Future Directions

**DOI:** 10.3390/ijms222312795

**Published:** 2021-11-26

**Authors:** Wojciech Grodzicki, Katarzyna Dziendzikowska, Joanna Gromadzka-Ostrowska, Marcin Kruszewski

**Affiliations:** 1 Chair of Nutrition Physiology, Department of Dietetics, Institute of Human Nutrition Sciences, Warsaw University of Life Sciences, 02-787 Warsaw, Poland; wojciech_grodzicki@sggw.edu.pl (W.G.); joanna_gromadzka_ostrowska@sggw.edu.pl (J.G.-O.); 2 Centre for Radiobiology and Biological Dosimetry, Institute of Nuclear Chemistry and Technology, 03-195 Warsaw, Poland; m.kruszewski@ichtj.waw.pl; 3 Department of Molecular Biology and Translational Research, Institute of Rural Health, 20-090 Lublin, Poland

**Keywords:** nanoplastic accumulation, inflammation, microbiome, intestinal barrier permeability, oxidative stress, neurotoxicity

## Abstract

The widespread usage of plastic places a significant burden on the environment and impacts numerous aquatic and terrestrial species. Humans in particular can be affected by plastic pollution, predominantly via inhalation and ingestion, as well as trophic transfer along the food chain. Under natural conditions synthetic materials undergo degradation into micro- and nanoparticles, especially prone to interact with biological systems. Organisms exposed to nanoplastic accumulate it in multiple tissues, including the gut and the brain. This phenomenon raises a question about the impact of nanoparticulate plastics on the communication pathways between these organs. The aim of this review is to explore an unsettling possibility of the influence of nanoplastic on the gut-brain axis and provide a comprehensive summary of available data regarding this subject. The scarce but consistent evidence shows that exposure to plastic nanoparticles can indeed affect both the digestive and the nervous system. Reported outcomes include microbiota alterations, intestinal barrier permeability, oxidative stress, inflammation, neurotoxicity and behavioral disturbances. Taking into consideration these alarming observations and the ubiquitous presence of plastics in human environment, more research is urgently needed in order to identify any potential threats that nanoplastic exposure can pose to the functioning of the gut-brain axis.

## 1. Introduction

The current era of the Earth’s history is frequently referred to as the Plasticene, the “Plastic Age”. Although coined informally, the term seems to appropriately reflect the state of the global environment, ubiquitously polluted with synthetic plastic litter [1]. During the last decades plastic production worldwide has been steadily increasing, reaching 368 million tons in 2019 [2,3]. The total amount of plastic ever produced by humans has been estimated to be more than 8 billion tons. Approximately 60% of that amount was discarded as waste and accumulated in the environment [4]. This rejected, unrecycled material not only contaminates the land, but also ends up in the aquatic biome, forming plastic debris both on and beneath the water surface [2]. In fact, a calculation made in 2014 estimated that over five trillion plastic fragments float in seas and oceans around the world [5]. This enormous number becomes even more overwhelming taking into consideration the fact that a substantial part of the synthetic waste consists of particles in microscopic or smaller scale, more prone to interact directly with biological systems [2,5]. Due to physicochemical and biological processes, such as UV-induced decomposition and digestion by marine species, under environmental conditions plastic can undergo degradation into micro- (defined as smaller than 5 mm in diameter) and nanoparticles (defined as smaller than 1000 or 100 nm in diameter). Compared with larger fragments, these micro- and nanoplastics (MNPs) pose a less tangible, but not less dangerous threat to organisms. As it turns out, they can be harmful especially in regards to digestive and nervous systems of aquatic organisms and other elements of the food chain, probably also including humans [2,6,7,8].

## 2. Plastics in Human Environment

Plastics are one of the materials most commonly used by humans, both in industry and everyday life. Their basic structure consists of repeated units forming polymers or copolymers that may be cross-linked or branched. Physicochemical properties of plastic depend on its chemical composition, especially on the inclusion of other chemical monomers that provide specific useful properties. The most prevalent types of plastic are polystyrene (PS), polyethylene (PE), polypropylene (PPL), polyvinyl chloride (PVC) and polyethylene terephthalate (PET). PS can be found in food containers and toys, PE in bin bags, shopping bags, bottle caps and bottles, PPL in straws and tubes, while PVC is commonly used in pipes or doors and PET is the main material in clothing fibers as well as containers for liquids and foods [9]. Plastics in the micro- and nanoparticulate form are pollutants that, like the original materials they come from, are chemically heterogeneous. Their chemical composition is very diverse, with the vast majority being PPL, PE and PS, followed by nylon, polyester and acrylic fibers. Statistically, polyurethane microparticles are the least common type of MNPs [10].

Plastic pollution, including MNPs, affects multiple environmental compartments. Given the fact that synthetic materials, by definition, are of human origin, the contamination starts on land through waste generation [9,11]. Marine ecosystems are particularly vulnerable to the negative impact of MNPs, as nearly 10% of the plastic produced annually is transported to seas and oceans, mostly via rivers. Moreover, especially small particles can also be easily spread by wind, making atmospheric air an important vehicle for MNPs distribution [9,12]. Different contamination sources raise questions about human exposure routes. Plastic can enter the body through three major pathways: by skin contact, inhalation and oral ingestion [13]. Dermal exposure results primarily from the use of hygiene products and clothing. Although this route is probably not a significant source of MNPs, since they do not penetrate deeply into skin layers, the available data are still not conclusive [13,14,15]. Regarding the exposure via respiratory tract, analyses of the atmospheric fallout and direct measurements of suspended particulate matter from different sources confirmed the presence of synthetic materials in the air [16,17,18]. Notably, concentrations of plastics are greater in suburban than urban areas and indoors compared to outdoors [16,17]. The total daily exposure calculated by Prata is estimated to fall within the range of 26 to 130 plastic microparticles/day and could be of physiological relevance [18].

In light of the existing data it seems that the main route of MNPs’ entry into the human body may be the gastrointestinal tract (GI tract). Therefore, special attention has been paid to the oral intake of MNPs, which seems to reach potentially harmful levels [19,20,21]. In a recent systematic review and meta-analysis Danopoulos et al. stated that the primary source of plastics in the human diet is seafood, which is not surprising, given the widespread contamination of aquatic environments [20]. In another review, Toussaint et al. found that up to 200 edible marine species can be affected by MNPs pollution, among which the content of plastic particles in sardines and sprats is especially well-documented [22]. Danopoulos et al. indicated that human exposure rate to MNPs could vary considerably depending on the geographic region, which determines to a substantial extent seafood consumption level. Based on the data available from the Food and Agriculture Organization of the United Nations (FAO), the authors calculated that the annual MNPs intake originated from different marine species combined could reach almost 54,000 particles per person, which equals 147.9 particles/person/day [20].

**Figure 1:**
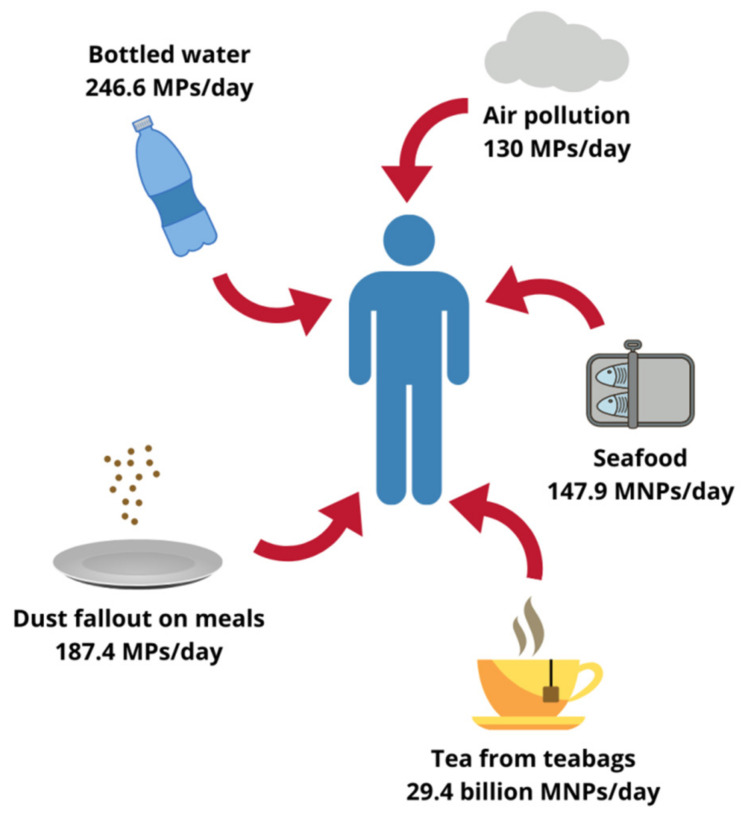
Possible human exposure level to plastics originating from different sources. MPs, microplastic particles; MNPs, micro- and nanoplastic particles. Designed using elements by ©Canva, sparklestroke, Pixeden, iconsy, OpenClipart-Vectors via Canva.com ( access date: 18 November 2021, version used Canva 2.0).

Although less evident, foodstuffs other than seafood can also contribute to MNPs oral exposure. Microscopic plastics have been found in honey, sugar and salt samples, the total content ranging from less than 10 to several hundred pieces/kg. Analyses of different beverages yielded similar results, beer being the least and bottled drinking water the most contaminated with particulate plastic [11,22]. A 2019 study by Hernandez et al. suggests that the list of relevant food sources of MNPs should also include tea. An experiment carried out on plastic teabags showed that under standard brewing conditions one cup of such a beverage could contain up to 2.3 million of microplastic particles (MPs) and 14.7 billion of nanoplastic particles (NPs). Therefore, only two cups of tea per day would expose the drinker to 29.4 billion MNPs, which far exceeds the amounts reported for any other food [19]. Moreover, some authors hypothesized that the aforementioned indoor air pollution could contribute to the oral exposure through dust settling on plates during meal preparation, making sensible estimations even more complicated. In fact, up to 68,415 MPs annually or 187.4 MPs daily could enter the human body this way [23]. According to Cox et al. the total microplastic consumption in the American population, inhalation and oral exposure combined, could surpass 121,000 particles/person/year. Drinking water alone, assuming only bottled one was consumed, could contribute to the ingestion of 90,000 MPs every year (or 246.6 MPs every day). Nevertheless, as emphasized by the authors, those values represent only a rough approximation and the real amounts might be higher [24]. As far as the actual mass of MNPs ingested by humans is concerned, Senathirajah et al. determined it to fall within the range of 0.1 to even 5 g/person/week. The authors based their estimations on an average mass of an individual plastic particle derived from different studies and took into consideration oral intake from shellfish, salt, beer and drinking water [25]. As a simple calculation shows, assuming a typical body weight of 70 kg, the level of exposure they reported translates into 0.2–10.2 mg/kg bw/day, which equals dosages used in studies of plastic toxicity [7]. Although staggering, this global average still does not reflect a whole picture, since it excludes many potential sources, such as inhalation route, and does not account neither for individual variability between consumers nor for different plastic characteristics [25]. Approximated levels of human plastic exposure from selected sources are summarized in Figure 1.

## 3. Toxic Potential of Plastic Particles

The toxic potential of MNPs is an open question. The literature published so far suggests that any severe, acute outcomes related to plastic exposure in humans are not likely. However, little is known about long-lasting, more subtle effects [8]. An important hint is the finding that MNPs are able to accumulate in different tissues. Studies on marine species showed that after exposure plastic particles were detected not only in the digestive tract and gills, the main routes of entry to the organism, but also in blood, liver, pancreas, heart and, notably, in the brain [7,8]. MNPs presence in biological compartments was associated with adverse effects, such as intestinal damage, shifts in microbiota composition, increased oxidative stress, inflammation, neuronal dysfunction and abnormal behavior [7,8,15].

Although much more scarce, experiments concerning mammalian models yield comparable outcomes. A recent review by Yong et al. identified four studies on rodents reporting MNPs accumulation. Following exposure, multiple pathological processes were observed, including gut dysbiosis, intestinal barrier dysfunction, changes in metabolism, increased oxidative stress and signs of neurotoxicity [8]. Additionally, in vitro experiments on various human cell lines showed that MNPs elicited oxidative stress, inflammatory response and cytotoxicity [8,15]. The literature directly related to human subjects is almost non-existing. Schwabl et al. detected MPs in stool of adult volunteers, suggesting MNPs impact on the digestive tract, whereas Ragusa et al. reported the presence of plastic particles in maternal placenta [26,27]. Taken together, the available data provide evidence for toxic potential of plastic particles, especially in the nanoscale [15]. Of note, their harmful effects are more pronounced in the digestive system, which is expected to be the major route of exposure. Moreover, a disturbing pattern of neurotoxicity emerges consistently as a severe side effect of NPs exposure among experiments conducted both in vitro and in vivo.

## 4. The Gut-Brain Axis

The GI tract and the central nervous system (CNS) are connected by a complex network of mutual interactions, known as the gut-brain axis (GB axis) [28]. The fact that the GI tract not only serves the role of digesting and absorbing nutrients, but also constitutes one of the main areas of contact between the organism and its external environment, makes it at the same time essential for survival and vulnerable to potential threats from outside the body. From this point of view it is logical that the alimentary canal is closely tied to the brain, the master controller of most physiological functions [29,30]. Noteworthy, an increasing body of evidence points towards an important role of microbiota in the bidirectional interplay between the gut and the brain [31,32,33].

The core of the GB axis consists of the vagus nerve, the X cranial nerve and simultaneously a branch of the autonomic nervous system (ANS). It sends information about the state of the inner organs, via afferent fibers, to the brain and connects the CNS to the enteric nervous system (ENS). The vagus nerve is sensible to diverse stimuli, including mechanical tension, hormones and other chemical incentives, that elicit a wide array of effects in the brain, stimulating regions related to feeding behavior, anxiety or emotions [28,31,33]. On the other hand, efferent vagal activity has an impact on the gut environment, influencing the immune system and metabolism [31,33]. Simultaneously, the CNS interacts with the GI tract via the hypothalamic-pituitary-adrenal axis (HPA axis), also integrated into the gut-brain communication pathways [31]. In response to stressors the hypothalamus releases corticoliberin, which stimulates the pituitary gland to produce adrenocorticotropin (ACTH). Subsequently, the ACTH acts on the adrenal glands, inducing the synthesis of cortisol (or corticosterone in rodents), the main stress hormone, able to modulate diverse gastrointestinal processes [28].

Direct effects of diverse neuronal and hormonal stimulation on the GI tract are possible due to the ENS, an intricate complex of nerves situated beneath the intestinal mucosa [31,33]. ENS is highly sensitive to different stimuli from the intestinal lumen and, given its connection to the vagus nerve, plays an important role in the communication of the GI tract with the CNS, sometimes being even referred to as the body’s “second brain” [28,31,33]. The plexuses of the ENS are located in close vicinity to the epithelium, which creates a tight barrier between the gut lumen and underlying tissues, preventing the unwanted passage of food and microbial antigens deep into the mucosa. A disruption of the barrier has been observed in several psychiatric disorders, including anxiety and depression, which suggests a link between gut permeability and CNS function [29,33]. Moreover, the intestinal barrier can be influenced by the HPA axis through cortisol activity and, on the other hand, by the gut microbiota. Microorganisms residing in the GI tract promote production of protective mucus and short-chain fatty acids (SCFA), which restore the intestinal epithelial cells and also the integrity of tight junctions [28,31,33]. Besides the physical defense provided by the intestinal barrier, the digestive tract is protected against multiple environmental factors by immune cells in the gut-associated lymphoid tissue (GALT) [29,31,34]. Specialized GALT components, namely M cells and dendritic cells, interact with antigens present in the lumen and stimulate the underlying T and B cells, situated in Peyer’s patches, to release cytokines. Those, in turn, induce immune response, which can propagate further outside the GI tract, reach the CNS via bloodstream and influence the vagus nerve signaling [29,31].

GALT contains the highest concentration of immune cells in the entire human organism and provides the primary space of exposure to microbial agents and their metabolites [31,34]. The totality of these microorganisms is known as the gut microbiota and, as a growing body of evidence demonstrates, heavily affects CNS functioning, blood-brain barrier (BBB) permeability, brain cells development and neuron maturation [31,32,34,35]. Additionally, microbial metabolites, such as tryptophan precursors, serotonin or catecholamines, act as neuromodulators, while SCFA produced by gut bacteria might stimulate the vagus nerve, affect neurotransmitter metabolism and have an impact on behavior [31]. Moreover, changes in microbiota have been linked to the development of different CNS-related disorders, including pathogeneses of Alzheimer’s disease, Parkinson’s disease, autism and depression [33].

Taken together, there are multifarious ways of communication between the gut and the brain. They entail diverse systems, such as the ANS, HPA axis, ENS, intestinal barrier, GALT and the microbiota, which maintain a constant dialogue and exchange information across different pathways encompassed by the GB axis [28]. Given the crucial physiological function of the axis and its involvement in numerous neurological disorders, any potential disrupting agents should be closely investigated. Therefore, the scarce but consistent evidence linking NPs exposure to both gut and brain alterations should be a cause of particular concern and requires thorough analysis.

## 5. Impact of Nanoplastic Exposure on the Gut-Brain Axis

Data regarding NPs and their impact on living organisms indicate that the main risk associated with plastic exposure is a non-acute toxicity, particularly with respect to the digestive and the nervous system [7,8]. The vulnerability of the GI tract can be explained by the fact that it constitutes one of the principal routes of entry for NPs into the organism and is subjected to a relatively high plastic load [19,24,36]. In regards to the CNS-related structures, there is a parallelism between the effects provoked by NPs and other nanoparticles. Many nanomaterials, especially metallic nanoparticles, have well-documented neurotoxic properties [37,38]. Moreover, evidence from human studies shows that nanoparticulate pollutants, such as combustion-derived nanoparticles, are able to accumulate in the vagus nerve, the core of the GB axis, and contribute to neurodegeneration [39]. Therefore, NPs of similar size can be expected to produce analogous outcome. Although, to the best of our knowledge, there are no studies directly focused on the impact of NPs on the GB axis, the available data provide hints about possible consequences of plastic exposure for the communication pathway connecting the GI tract and the brain. The following is the description of experimental studies investigating NPs-related toxicity in regards to several elements of the GB axis.

### 5.1. In Vitro Studies on Cellular Cultures

Preliminary findings from several in vitro experiments yield noteworthy results regarding NPs impact on different cell lines originating from the GI tract and the CNS. Busch et al. investigated the effects of 59 nm pristine polystyrene (PS) particles, 59 nm amino-modified PS NPs or 279 nm PVC NPs (1–50 µg/mL, 24 h) in a cellular model of healthy and inflamed human intestine using epithelial (Caco2 and HT29-MTX-E12) and immune cells (THP1 macrophages). The treatment of monocultures with amino-modified PS NPs caused a dose-dependent reduction of metabolic activity, inflammatory response evidenced by an increased release of interleukin 1β (IL-1β) and DNA damage. None of these were present if pristine PS or PVC NPs were applied. In the triple culture models, in healthy cells treated with modified PS the authors reported increased cytotoxicity and decreased expression of tight junction protein 1, suggesting compromised intestinal barrier integrity. They also observed a dose-dependent loss of nuclei in the epithelial layer in the inflammation model treated with PVC, but not with modified or non-modified PS. The most pronounced effects were found for amino-modified PS NPs, known to destabilize cell membranes and disintegrate lysosomes upon intracellular uptake. However, these data are not relevant to the environmental exposure, since these NPs are used only in experimental conditions. Nevertheless, the slight toxicity induced by PVC NPs means that they could potentially aggravate an already existing inflammatory state [40].

In a similar experiment Domenech et al. studied the effects of PS nanoplastic (5–100 nm, 1–100 µg/mL) exposure on human intestinal barrier models (Caco-2/HT29 and Caco-2/HT29 + Raji-B cells). In line with previous results no significant changes in the expression of oxidative stress-related genes and no signs of toxicity were observed after 24 h of treatment [41]. Hesler et al. also did not report cytotoxicity of COOH-modified PS NPs (50 nm or 500 nm), applied for 24 h (0.1–100 µg/mL) to an intestinal Caco-2/HT29-MTX-E12 co-culture model of the intestine, despite a marked PS NPs uptake by intestinal cells. The authors hypothesized that this intracellular accumulation could result in long-term toxicity and distribution to other tissues [42]. Indeed, translocation of NPs was reported by Walczak et al., who compared differently charged non-digested and artificially pre-digested PS NPs (50 nm, 250 µg/mL, 24 h) in the same co-culture intestinal model. Interestingly, pre-digestion process significantly increased NPs translocation through intestinal cell layer, which is highly relevant for real-life conditions of oral plastic exposure. Moreover, positively charged pre-digested NPs provoked a decrease of intestinal barrier integrity, measured by transepithelial electrical resistance, and increased its permeability [43].

NPs have also been studied in regards to their impact on brain cells. Jung et al. treated different murine cells with 100 nm PS NPs and observed intracellular deposition of NPs in astrocytes as well as in mixed neuronal culture, especially in cell bodies of neurons close to the nuclei. Neuronal cells were also affected in terms of viability, which was significantly reduced due to the NPs treatment. Nanoplastic also influenced neuronal development, as shown by alterations in the expression of genes involved in the process, namely *Tubb3* and *Gfap*. In addition, in astrocytes PS NPs increased proinflammatory signaling, including up-regulation of the tumor necrosis factor alpha (TNF-α) gene (*Tnfa*) and IL-1β (*Il1b*). Although the viability of astrocytes was not affected by PS NPs, the authors speculated that the exposure might have induced reactive astrocytosis, leading to inflammation and death of nearby neurons [44]. Signs of neurotoxicity were also reported by Schirinzi et al., who explored cellular effects caused by different kinds of MNPs, including PE micro- (3–16 µm) and nanoparticles (100–600 nm) as well as PS micro- (10 µm) and nanoparticles (40–250 nm). Treatment of human glioblastoma T98G cells with plastics for 24 h resulted in an increased generation of reactive oxygen species (ROS), the effects being more noticeable for PS [45].

In another study Murali et al. sought to investigate in vitro reactivity of “fresh” or “aged” PS NPs (45–70 nm) with distinct surface modifications and in conjunction with microbial toxins. In the case of “fresh” NPs the authors did not observed any uptake of NPs by neurons or astrocytes, however, intracellular accumulation occurred in microglia exposed to carboxylated particles. These NPs were found to be severely toxic at the highest concentrations, in contrast to the ones coated with polyethylene glycol that turned out to be harmless. Interestingly, assays involving NPs after 6 months storage brought very different results. The process of ageing not only enhanced the cellular uptake of NPs, possibly through the endocytotic pathway, but also increased their cytotoxicity in NE-4C neuronal stem cells and microglia. Furthermore, the authors observed that bacterial lipopolysaccharide (LPS) was easily adsorbed by NPs and promoted their uptake by microglia cells, which could lead to increased cytotoxicity [46]. These important findings show that, while non-modified plastic nanoparticles may be relatively innocuous, changes they likely undergo in the environment can significantly alter their properties.

Results confirming nanoplastic neurotoxicity in vitro were also described by Hoelting et al., who employed an embryonic stem cell (hESC)-derived 3-dimensional model of human neural development exposed to 33 nm PE NPs in a short- and a long-term fashion. In the short term (48 h) the majority of neurospheres internalized NPs only when exposed to the highest concentration that resulted in cytotoxicity and oxidative stress, evidenced by an increase of malondialdehyde. After 18 days of treatment, however, accumulation of nanoplastic was detectable even at low concentrations. Although non-toxic, low concentration down-regulated genes related to neurodevelopment, such as *HES5*, *NOTCH1*, *FOXG1*, *NEUROD1* and *ASCL1*. The authors concluded that their findings highlight the possibility of NPs-induced developmental neurotoxicity [47].

Currently, in vitro research exploring the toxicity of NPs with regards to the GI tract and CNS cells is still scarce. In addition, in the limited number of existing studies different types, sizes and concentrations of nanoplastics have been used, which complicates direct comparisons. Nevertheless, those preliminary findings share certain commonalities, allowing for some generalization. NPs seem to be able to penetrate gut cells, creating an opportunity for further distribution. In brain cells models nanoplastic is internalized, elicits oxidative stress and reduces cell viability, particularly in the more realistic, “aged” form. The summary of these research is presented in Table 1.

### 5.2. In Vivo Studies on Fish

A relatively high number of studies have investigated the impact of MNPs on marine organisms, perhaps the most striking being the one describing a recently discovered new crustacean species, *Eurythenes plasticus*, whose name refers to the plastic microfiber found within the animal’s alimentary canal [48]. Nonetheless, in regards to the NPs-induced effects relevant to the GB axis, published articles regarding in vivo experiments on aquatic vertebrates species are not abundant. Veneman et al. studied NPs impact on transcriptomic responses in zebrafish larvae (*Danio rerio*). 700 nm PS NPs (5 mg/mL) were injected into 2-day old embryos resulting in multiple changes in gene expression, though NPs did not penetrate beyond the vascular system. The exposed larvae showed alterations in the expression of 26 genes 1-day post-injection and 51 genes 3 days after the treatment, showing signs of complement system stimulation as well as activation of pathways related to toxicity and oxidative stress [49].

Similarly, changes in ROS and antioxidant parameters were observed upon exposure to PS nano- (50 nm) and microparticles (45 µm) of Marine medaka (*Oryzias melastigma*). Histological analysis showed that nanoplastic particles accumulated within and around digestive organs. Moreover, nanoplastic, but not microplastic, increased the activity of antioxidant enzymes, superoxide dismutase (SOD) and catalase (CAT), in the gut and induced apoptosis detected as DNA breaks. However, microparticulate PS affected microbiota composition more significantly than nanoparticulate PS, mainly by decreasing the abundance of *Bacteroidetes* [50]. These results indicate that the effects provoked by MNPs are size-specific and that NPs may be more prone to disturb the redox balance, while MPs cause a more pronounced dysbiosis.

Fish vulnerability to NPs was also studied by Kashiwada, who exposed adult Japanese medaka (*Oryzias latipes*) to 39.4 nm PS NPs, which resulted in a significant accumulation of NPs in the intestine as well as in the brain, indicating their ability to cross the BBB. Unfortunately, no further assays were performed, apart from the distribution patterns, making it impossible to draw conclusions regarding any potential neurotoxic effects [51]. In line with these results, accumulation of PS NPs (51 nm) was detected by Pitt et al. in the GI tract and head of *Danio rerio* embryos treated from 6 to 120 h post-fertilization. Although no acute toxicity was observed, behavioral tests revealed reduction of larval locomotor activity, suggesting NPs deposition in the brain. These findings indirectly confirm the harmful effect NPs could have had on the developing CNS [52].

An interesting approach to the NPs toxicity involving the food chain effects was described by Chae et al., who studied the trophic transport and toxicity of 51 nm PS NPs on four different marine species. First, green algae (*Chlamydomonas reinhardtii*) were exposed to NPs for 72 h, then the algae were given to planktonic neonates (*Daphnia magna*) for 5 h, which in turn served as feed for Chinese medaka (*Oryzias sinensis*) for 48 h. Finally, *O. sinensis* were given to a predator fish, dark chub (*Zacco temminckii*) for 24 h. Screening for NPs accumulation performed after the food chain-mimicking procedure revealed penetration of PS NPs into the algae cells and their presence in the gut of *D. magna*. The accumulation of PS was accompanied by histological changes visualized as damage to the intestinal wall. In final consumers nanoplastic was detected in the gut of *O. sinensis* and in both the gut and the stomach of *Z. temminckii*. Thus, the trophic transfer potential of NPs in the aquatic environment was confirmed. Furthermore, every species was exposed to NPs individually to evaluate the toxicity separately. No significant effects were reported neither for *C. reinhardtii* after treatment for 72 h nor for *D. magna* exposed for 48 h. However, harmful impact of nanoscale plastic became more evident in the vertebrates undergoing a 7-day treatment. Both *O. sinensis* and *Z. temminckii* showed disturbed locomotive activities, manifested as abnormal swimming patterns. These behavioral changes, as the authors hypothesized, could have been related to brain damage caused by the exposure [53].

Another study involving trophic transfer of NPs in aquatic environment was conducted by Mattsson et al., who investigated the effects of sulfonated 24 and 27 nm PS NPs delivered through a 3-level food chain to the Crucian carp (*Carassius carassius*). First, nanoplastic was given to algae (*Scenedesmus* sp.), which were later eaten by zooplankton (*D. magna*). Finally, the contaminated *D. magna* served as food for the carps. Behavioral tests conducted after 61 days of treatment showed slower movement, less exploration activity and diminished hunting performance in carps receiving nanoparticles compared to the control group. Moreover, histological analysis revealed changes in color, texture and water content of brains sampled from exposed fish, suggesting an impact of NPs. The authors explained these findings by the affinity of PS to lipid molecules, resulting in a possible accumulation in fat-rich organs, such as the brain [54]. These results were later corroborated by the same authors, who in a similar experiment investigated the effects of 180 nm and 53 nm amino-modified PS delivered to fish through an analogous food chain. As observations revealed, carps exposed to 53 nm NPs ate more slowly and swam longer before reaching the prey. These effects, affecting feeding efficiency and potentially undermining environmental survival, were linked to alterations in the brain. Cerebral tissue of exposed fish not only accumulated nanoplastic, but also contained less water and underwent morphological changes in the gyri of cerebral lobes [55].

A deeper insight into neurotoxic mechanisms of NPs was provided in the study by Chen et al., who examined the influence of PS MPs (45 µm) and NPs (50 nm) on zebrafish larvae behavior, gene expression and enzymatic activity. Treatment with both types of particles resulted in size-dependent alterations in exposed embryos. Whereas swimming distance in darkness was not altered by MPs, treatment with NPs reduced it notably. Principal component analysis showed that these behavioral alterations were accompanied by changes in antioxidant biomarkers, among which reduced glutathione (GSH) level was significantly lowered in comparison with the control group. Exposure to nanoplastic also affected body length, which was found to be decreased in exposed larvae. In addition, two genes involved in neurodevelopment, namely *Gfap* and *α1-tubulin*, were up-regulated only in the NPs group. Finally, the activity of acetylcholinesterase (AChE), a key enzyme involved in neurotransmission, was significantly decreased due to the NPs, but not the MPs treatment [56].

Similarly unsettling outcomes were reported by Sarasamma et al. in an in vivo study on adult zebrafish treated with 70 nm PS NPs in acute (7 days) or chronic (30 days or 7 weeks) regimes. Histological analysis after the 30-day exposure period revealed that plastic particles were present in multiple tissues, including the intestine and the brain. Moreover, detailed analyses of brain tissues sampled after the 7-day experiment showed a decrease in numerous neurotransmitter and hormone levels. Alterations included lowered AChE, dopamine, melatonin, γ-aminobutyric acid, 5-hydroxytryptophan, vasopressin, kisspeptin and oxytocin. In terms of behavior, 7-week exposure to NPs led to circadian dysregulation in terms of locomotive activity, including a reduction in average speed and rapid movement time ratio during the light cycle as well as hypoactivity during the dark cycle. The 7-day treatment resulted in exploratory hyperactivity, reduced aggressiveness, worsened predator avoidance and altered shoaling. Notably, disturbances in most of the behavioral endpoints measured were more pronounced in the group exposed to the higher concentration of NPs [57]. This thorough behavioral assessment, together with the confirmation of tissue distribution and changes in the CNS, provide a strong evidence for NPs-induced neurotoxicity and highlight the importance of plastic load, with higher concentrations being significantly more harmful.

Data regarding NPs impact on GB axis derived from in vivo studies on aquatic vertebrates are still insufficient to draw definitive conclusions. Furthermore, the size and concentration of particles applied are often too dissimilar to confidently compare results derived from different experiments. However, studies conducted up to date are consistent in at least several aspects. They clearly show size-dependent differences in toxicity, NPs being more harmful than MPs, possibly due to their smaller diameter and, consequently, higher bioactivity. There is also convincing preliminary evidence for translocation of NPs from the gut to the brain and their ability to cross the BBB. In conjunction with behavioral alterations, possible neurodevelopmental disturbances, impact on enzymatic activity, induction of oxidative stress and immune system activation, the influence of nanoplastic on the GB axis becomes a plausible phenomenon. The summary of these research is presented in Table 2.

### 5.3. In Vivo Studies on Rodents

Research into plastic toxicity in mammalian species is currently very scarce and in most part focused on the effects caused by MPs. Consequently, studies investigating nanoplastic effects in regards to the GB axis are even more lacking. In fact, a recent review by Yong et al. mentioned only 10 articles describing MNPs effects in mice, whereas another review by Prüst et al. identified only one such publication directly related to NPs neurotoxicity [7,8]. Nevertheless, the existing evidence allows to formulate some initial remarks and is definitely worth exploring. 

One of the few studies that examined in vivo effects of NPs in rodents was conducted by Walczak et al. as a continuation of their already mentioned in vitro experiments. The authors employed different types of 50 nm PS NPs, either pristine, positively or negatively charged. Plastics were administered orally to 5-week-old male Fischer rats as a single dose of 125 mg/kg bw. As subsequent analyses revealed, NPs accumulated in the intestinal wall and were further distributed to other tissues, such as kidneys, spleen, testis or heart. However, the degree of accumulation varied depending on surface modification of particles, the most pronounced effects being observed in the case of negatively charged NPs. Their calculated bioavailability was also much higher, reaching 1.5–1.7% compared to only 0.2–0.3% for pristine or positively charged particles [58]. These results clearly demonstrate the ability of NPs to affect the digestive system and other tissues, but also indicate that surface modification may significantly influence nanoplastic-induced effects in living organisms.

Limited data indicate that NPs could accumulate in mammalian organisms also in the CNS. Reineke et al. exposed male Sprague-Dawley rats orally to PS particles, 500 and 1000 nm in size, for 5 h. Primary observations revealed greater amounts of 500 nm NPs in the stomach, whereas more 1000 nm MPs were found within the intestine. While MNPs of both sizes were present mainly in the liver, kidneys and lungs, the smaller ones, and only them, were also detected in the brain. An important finding was also the observation that the uptake rate was inversely correlated with particle size, suggesting that 500 nm NPs penetrated internal body compartments easier than larger particles [59]. Further insight into NPs distribution was provided by Fournier et al., who treated pregnant Sprague-Dawley rats with a single dose of 20 nm PS NPs via inhalation. The analysis of tissues collected from the exposed animals showed that NPs accumulated in maternal lungs, heart, spleen and uterus, but also in the placenta, fetal liver and heart. Subsequent microscopic examination revealed nanoplastic presence in lungs, kidneys and brains of fetuses. An additional ex vivo experiments on isolated placentas confirmed the results obtained in vivo. Thus, transmission of NPs to the fetus during pregnancy was proved to occur [60]. These results corroborate in vivo accumulation of NPs in the brain and highlight their potential for inducing neurodevelopmental toxicity.

While the aforementioned studies focused on NPs distribution, the subsequent experiment revealed direct impact of nanoplastic on several GI tract-related parameters. Lu et al. found that oral exposure of mice to 500 nm PS NPs and 50 µm MPs resulted in a decline in body weight and a decrease in gut mucin secretion, accompanied by a lowered expression of mucin-related genes, such as *Muc1* and *Klf4*. Additionally, numerous changes in mice intestinal microbiota were identified, in particular a significant decrease in *Firmicutes* bacteria. In animals treated with NPs 310 microbial operational taxonomic units (OTUs) differed from the control group, while in the MPs-exposed mice 160 OTUs were altered. As the authors concluded, MNPs of different sizes weakened the intestinal barrier and caused dysbiosis, which could eventually lead to further health problems [61]. Since microbial neuromodulatory metabolites can stimulate the vagus nerve and SCFA produced by gut bacteria are able to cross the BBB, it can be expected that any disturbances in the gut microbiota may affect brain physiology, both indirectly and directly [31,62], which, in turn, might result in behavioral disturbances.

An explicit attempt to investigate nanoplastic neurotoxicity was made by Rafiee et al., who performed behavioral tests on adult male Wistar rats exposed orally to 38.9 nm pristine PS nanoparticles for 35 days. To evaluate nanoplastic influence on locomotive activity, coordination, anxiety, avoidance and spatial working memory a battery of behavioral assessments was applied, however, the authors reported no statistically significant results. Only subtle changes in the exposed rats were observed [63]. Also, Xiao et al. investigated NPs impact on rodent behavior as well as microbiota composition, intestine and brain. Similarly, the authors did not observe any behavioral alterations in mice treated orally with PS NPs (around 50 nm) for 30 days. Neither inflammation nor oxidative stress parameters were affected in murine GI tract and brain, even though the intestinal wall was slightly damaged in the group exposed to the highest NPs concentration (10 mg/kg bw). Additionally, microbiota composition was altered in terms of β-diversity [64].

However, more pronounced nanoplastic toxicity has been reported in a recent study by Xu et al., who administered 100 nm PS NPs (pristine, carboxyl- or amino-modified) to mice for 28 days by oral gavage. The exposure provoked nanoplastic accumulation in several organs, including kidneys, testis, spleen, lungs, GI tract and the brain. Histological damage to target tissues was detected, including destruction of the epithelium in the intestine as well as neuron malformation in the cerebral cortex. Changes in the brain were accompanied by an increased expression of proinflammatory TNF-α and interleukin 6 (IL-6). The authors performed further in vitro experiments on human Caco-2 cells to provide a mechanistic insight into processes observed in vivo. These additional analyses confirmed that PS NPs infiltrated intestinal cells and disrupted the integrity of the intestinal barrier [65]. Summarized data of in vivo studies on rodents are presented in Table 3.

## 6. Main Findings

In spite of the long history of plastic usage and its ubiquitous presence in the environment, surprisingly little is known about the impact of synthetic materials on human health. Even though the number of studies investigating their interaction with biological systems systematically increases, the majority of questions remain vastly unexplored. One of the principal phenomena of concern in regards to plastic pollutants is their degradation into smaller forms of micro- and nanoparticles, among which especially the latter are hardly studied, although other nanomaterials have been shown to elicit a wide array of toxic effects. The data gathered so far draw a disturbing image of nanoplastic afflicting numerous species, including humans. Different exposure routes, predominantly ingestion and inhalation, further exacerbated by the trophic transfer across the food chain, make the potential health risks very plausible. The current evidence consistently points towards the ability of NPs to first accumulate in the digestive tract and later translocate to other tissues, including even the well-protected internal body compartments, such as the brain. The fact that nanoplastic can reach those two vital structures in particular suggests it may have an impact on communication pathways connecting the GI tract and the CNS.

**Figure 2:**
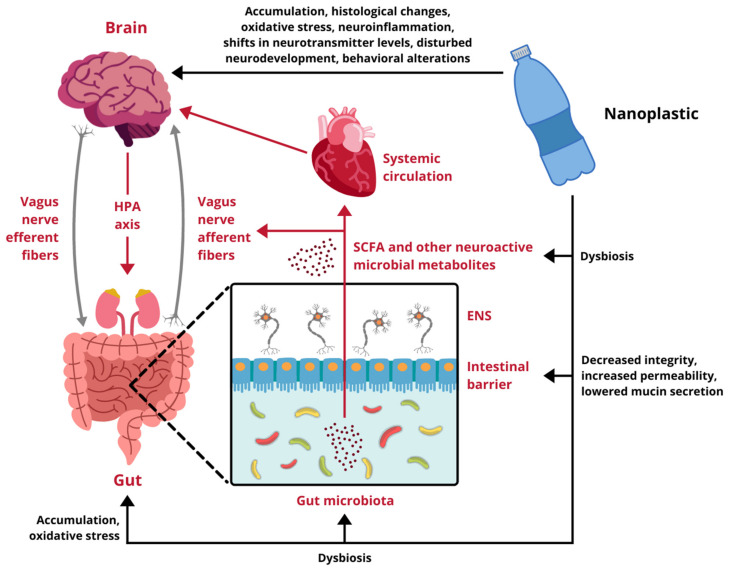
Impact of nanoplastic exposure on the gut-brain axis. HPA axis, hypothalamic-pituitary-adrenal axis; ENS, enteric nervous system; SCFA, short-chain fatty acids. Designed using elements by ©HaticeEROL, sparklestroke, sketchify, Twemoji, Sketchify Edu, Clker-Free-Vector-Images, iconsy via Canva.com ( access date: 18 November 2021, version used Canva 2.0).

Research regarding the influence of NPs on different components of the GB axis is scarce and only begins to scratch the surface of possible toxicity. Available data come exclusively from experiments performed on cellular cultures and animal models, therefore, any indications of potential risks for human health have to be extrapolated from these results. In vitro studies demonstrate that nanoscale plastic particles undergo internalization, both in intestinal and cerebral cells, provoking reduced viability and oxidative damage. Moreover, under environmentally realistic conditions, they are able to adsorb other toxins, which contribute to their harmfulness. In vivo experiments on aquatic vertebrates confirm these observations, proving NPs capable of effectively distribute over the body, affecting the digestive tract and the brain. They trigger the immune response, disturb the intestinal microbiota homeostasis, induce oxidative stress and cause behavioral alterations. Finally, the few studies conducted on rodents are in line with the aforementioned research and show several alarming effects taking place upon exposure to NPs. In mammals nanoplastic accumulates in the GI tract, induces dysbiosis and undermines the intestinal barrier integrity. Furthermore, it translocates to multiple organs and passes across biological barriers, including the placental-blood barrier and the BBB, to ultimately enter the brain. Summarized effects of NPs exposure on the GB axis derived from studies presented in this review are depicted in Figure 2.

## 7. Conclusions and Future Perspectives

Although the experimental data regarding nanoplastic impact on mammalian systems are just beginning to build up, the evidence gathered up to date sheds some light on the consequences NPs exposure could have for both the GI tract and the CNS. The accumulation in the digestive system seems to be a factual phenomenon that might lead to dysbiosis and jeopardize the integrity of the intestinal barrier. Further biodistribution of NPs also takes place, as their presence in multiple tissues is shown consistently. One of the target organs is the brain, which suggests that nanoparticulate plastic possesses the ability to cross the BBB. Even though specific behavioral or biochemical alterations in the CNS are yet to be proven, the fact that NPs can reach cerebral compartments and affect the gut environment opens up the alarming possibility of compromised functioning of the GB axis. Toxicity determinants, such as plastic type, particle size and load, surface modification or adsorption of chemicals, as well as impact on gene expression and specific biochemical pathways involved in the gut-brain communication are examples of topics that future investigation should aim to address. Regardless of the outcomes, the widespread plastic contamination in the human environment makes preventive measures and caution highly advisable.

## Figures and Tables

**Table 1:** In vitro NPs toxicity related to the GB axis.

Cell Models	NPs Type and Size	Exposure	Effects Related to the GB Axis	Reference
Human intestinal Caco-2, HT29-MTX-E12 and THP-1 monocul tures; triple culture human intestinal Caco-2/HT29-MTX-E12/THP-1 model(healthy or inflamed)	Pristine/amino-modified PS NPs (59 nm) PVC NPs (279 nm)	24 h (1–50 µg/mL)	Monocultures/amino-modified PS: metabolic disruption, inflammation, DNA damage; healthy triple culture model/amino-modified PS: increased cytotoxicity, decrease of tight junction protein 1; inflamed triple culture model/PVC: loss of nuclei	[40]
Human intestinal Caco-2/HT29 and Caco-2/HT29 + Raji-B cells	PS NPs (5–100 nm)	24 h (1–100 µg/mL)	No significant toxic effects	[41]
Human intestinal Caco-2/HT29-MTX-E12co-culture model	Carboxylated PS NPs (50 and 500 nm)	24 h (0.1–100 µg/mL)	Uptake of NPs	[42]
Human intestinal Caco-2/HT29-MTX co-culture model	Pristine/positively/negatively charged PS NPs (50 nm), non-digestedor digested in vitro	24 h (250 µg/mL)	Digested NPs: enhanced translocation across cells; positively charged NPs: increased intestinal barrier permeability	[43]
Murine mixed neuronal cells; primary astrocytes	PS NPs (100 nm)	48 h (50–200 µg/mL)	Uptake of NPs; mixed neuronal cells: reduced cell viability, altered expression of *Tubb3* and *Gfap*; primary astrocytes: increased expression of *Tnfa* and *Il1b*	[44]
Human neuronal T98G cells	PE NPs (100–600 nm) PS NPs (40–250 nm)	24 h (0.05–10 µg/mL)	Increased ROS generation	[45]
Murine NE-4C Neuroectodermal stem cells; neuron-enriched primary brain cell cultures; primary astrocytes; microglial cultures; brain vascular endothelial cell cultures	Carboxylated/PEGylated PS NPs (45–70 nm), “fresh” or “aged”(6 months < of storage)	1 h (50 µg/mL) 24 h (7.8–250 µg/mL)	“Fresh” carboxylated NPs: uptake by microglia; “aged” NPs: uptake and cytotoxicity in NE-4C neuronal stem cells and microglia; enhanced cellular uptake of NPs caused by LPS adsorption	[46]
Embryonic stem cell (hESC)-derived 3-dimensional model of human neural development	PE NPs (33 nm)	48 h (5.6–1440 µg/mL) 18 days (5.6–360 µg/mL)	Uptake of NPs; reduced cell viability; oxidative stress; down-regulation of *HES5*, *NOTCH1*, *FOXG1*, *NEUROD1* and *ASCL1*	[47]

**Table 2:** Summarized data derived from in vivo experiments on fish regarding toxic effects of NPs related to the GB axis.

Fish	NPs Type and Size	Exposure	Effects Related to the GB Axis	Reference
Zebrafish (*D. rerio*)	PS NPs (700 nm)	Single-dose injection (5 mg/mL)	Altered expression of 26 genes 1 day and 51 genes 3 days post-injection; activation of the complement system; activation of oxidative stress-related pathways	[49]
Marine medaka (*O. melastigma*)	PS NPs (50 nm)	In water for 24 h (10 µg/mL) or 14 days (2.5 µg/mL)	NPs accumulation in the digestive system; induction of apoptosis in the gut; increased activity of SOD and CAT in the gut	[50]
Japanese medaka (*O. latipes*)	PS NPs (39.4 nm)	In water for 7 days (10 µg/mL)	NPs accumulation in the gut and brain	[51]
Zebrafish (*D. rerio*)	PS NPs (51 nm)	In water for 114 h (0.1–10 µg/mL)	NPs accumulation in the gut and head; behavioral alterations	[52]
Chinese medaka (*O. sinensis*) Dark chub (*Z. temminckii*)	PS NPs (51 nm)	In water for 7 days (5 µg/mL, individual toxicity) For 48 h (*O. sinensis*) or 24 h (*Z. temminckii*) via trophic transfer (*C. reinhardtii* → *D. magna* → *O. sinensis* → *Z. temminckii*)	Individual toxicity: behavioral alterations; *O. sinensis*/trophic transfer: NPs accumulation in the gut; *Z. temminckii*/trophic transfer: NPs accumulation in the gut and stomach	[53]
Crucian carp (*C. carassius*)	Sulfonated PS NPs (24 and 27 nm)	For 61 days via trophic transfer(*Scenedesmus* sp. → *D. magna* → *C. carassius*)	Histological changes in the brain; behavioral alterations	[54]
Crucian carp (*C. carassius*)	Amino-modified PS NPs (53 and 180 nm)	For 67 days via trophic transfer(*Scenedesmus* sp. → *D. magna* → *C. carassius*)	NPs accumulation in the brain; behavioral alterations	[55]
Zebrafish (*D. rerio*)	PS NPs (50 nm)	In water for 117 h (1 µg/mL)	Up-regulation of *Gfap* and *α1-tubulin*; decreased AChE activity; decreased levels of GSH; decreased body length; behavioral alterations	[56]
Zebrafish (*D. rerio*)	PS NPs (70 nm)	7 days (0.5 and 1.5 µg/mL) 30 days (1.5 µg/mL) 7 weeks (5 µg/mL)	NPs accumulation in the gut and brain; lowered levels of AChE, dopamine, melatonin, vasopressin, 5-hydroxytryptophan, kisspeptin, γ-aminobutyric acid and oxytocin; behavioral alterations	[57]

**Table 3:** Summarized data derived from in vivo experiments on rodents regarding toxic effects of NPs related to the GB axis.

Rodent	NPs Type and Size	Exposure	Effects Related to the GB Axis	Reference
Fischer rat	Pristine/positively/negatively charged PS NPs (50 nm)	Single-dose orally (125 mg/kg bw)	NPs accumulation in the gut	[58]
Sprague-Dawley rat	PS NPs (500 nm)	Orally for 5 h (100–125 mg/kg bw)	Accumulation in the GI tract and brain	[59]
Sprague-Dawley rat (pregnant)	PS NPs (20 nm)	Single-dose inhalation (2.64 × 10^14^ particles)	NPs accumulation in fetal brain	[60]
ICR mouse	PS NPs (500 nm)	Orally in drinking water for 5 weeks (0.1 or 1 µg/mL)	Higher load: decreased body weight; decrease in gut mucin secretion; lowered expression of *Muc1* and *Klf4*; dysbiosis	[61]
Wistar rat	PS NPs (38.9 nm)	Orally for 35 days (1–10 mg/kg bw)	No changes in behavior	[63]
C57BL/6J mice	PS NPs (around 50 nm)	Orally for 30 days (0.2–10 mg/kg bw)	No changes in behavior; no inflammation/oxidative stress in the gut and brain; highest dose: damage to the intestinal wall; changes in microbiota composition	[64]
BALB/c mice	Pristine/carboxyl-/amino-modified PS NPs (100 nm)	Orally for 28 days (1 mg/day)	NPs accumulation in the gut and brain; histological damage to the gut and brain; inflammation in the brain; intestinal cells penetration confirmed in vitro	[65]
